# Comparative Evaluation of Surface Roughness and Color Stability Between Single-Shade Composite and Multi-Shade Composite: An In Vitro Study

**DOI:** 10.7759/cureus.65396

**Published:** 2024-07-25

**Authors:** Angela Alex, Vijay Venkatesh

**Affiliations:** 1 Conservative Dentistry and Endodontics, SRM Kattankulathur Dental College and Hospital, Chennai, IND

**Keywords:** single-shade composite, spectrum, omnichroma, surface roughness, color stability, toothbrush simulation, multi-shade composite

## Abstract

Introduction

Composite resin materials are a popular choice for direct tooth-colored restorative purposes due to their excellent aesthetic qualities and versatility. The key determinants that impact the visual aspect of the restoration are gloss, surface roughness, and color stability. Research indicates that there is a direct correlation between the level of roughness on a composite resin surface and the likelihood of discoloration. The aim of this study was to compare and evaluate the surface roughness and color stability of a single-shade and multi-shade composite resin after subjecting it to toothbrush simulation and immersion in coffee and an aerated drink.

Materials and methods

Ten single-shade composite resins and 10 multi-shade composite resins were packed into a Teflon mold and light cured. Pre-operative surface roughness values were evaluated using a surface profilometer. Toothbrush abrasion was simulated using a toothbrushing simulator. All these resin molds were then evaluated for initial color using a spectrophotometer machine (Konica Minolta, Japan). Five resin molds were then immersed in a beaker containing coffee for seven days and five resin molds were immersed into a beaker containing an aerated drink for seven days. Following this, the resin molds were re-evaluated for color stability using a spectrophotometer machine.

Results

The results of this study show that single-shade composite showed increased discoloration when compared with multi-shade composite resin. Also, there is a statistical difference between the single-shade composite and multi-shade composite when it comes to surface roughness and discoloration.

Conclusion

With the limitations of the present study, it can be concluded that single-shade composite resins have more discoloration potential in beverages than multi-shade composite resins.

## Introduction

For direct tooth-colored restorative purposes using composite resin materials, the key determinants that impact the visual aspect of the restoration are gloss, surface roughness, and color stability [1]. The surface roughness has a considerable impact on the gloss of composite materials. Surface roughness significantly influences the buildup of dental plaque and is a crucial element in the development of staining. For any cosmetic restorative material to be considered effective, it must accurately replicate the natural tooth color and also retain the color consistently over extended durations [2].

Research indicates that there is a direct correlation between the level of roughness on a composite resin surface and the likelihood of staining with subsequent discoloration. This might have a negative impact on the overall appearance of the restoration. Toothbrush abrasion in the oral cavity is particularly undesirable for composite restorations due to its negative impact on aesthetics and biology, resulting in reduced gloss and discoloration [2].

Color alterations have been linked to dietary factors, chemical interactions, the uptake of water, and inadequate oral hygiene [3]. The consumption of specific beverages can impact the aesthetic and physical characteristics of resin composites, leading to a decline in the quality of restorations [4]. Resin-based restorative materials can be discolored to varying degrees by beverages such as coffee, aerated drinks, and wine [5].

Applying nanotechnology to the field of dentistry, producers are currently offering resin composite materials that utilize single-shade schemes as opposed to more complex color systems [6]. The product exhibits unique attributes derived from the implementation of "Smart Chromatic Technology." The tooth has the potential to capture the structural color of its surroundings, which is determined by the size of its nanofiller particles. The product is devoid of any additional dyes or pigments. However, the fillers themselves generate a structural color that ranges from red to yellow, matching the color of the adjacent teeth. The smart monochromatic composite is a single-color material designed to perfectly match all 16 VITA classical shades [7]. The benefit of its color-matching capability is that it obviates the requirement for shade-taking before the procedure [6]. Omnichroma (Tokuyama Dental America) is a single-shade composite, which utilizes structural color with its 260 nm spherical fillers [7]. Whereas, spectrum (Dentsply Sirona) is a multi-shade composite, which utilizes red and yellow colorants to accurately match tooth colors [8].

Since the color stability and surface roughness of composite resins are crucial factors for achieving aesthetic success, the aim of this study was to assess the surface roughness of a single-shade composite compared to a multi-shade composite, following exposure to a toothbrush simulator [3]. Additionally, the study aimed to evaluate the color stability of the composites after immersing them in coffee and an aerated drink (coca-cola) for a duration of one week.

## Materials and methods

This study was approved by the Institutional Research Ethics Committee at the SRM Medical College Hospital and Research Centre (ethical clearance number: SRMIEC-ST1123-1264)

Sample size calculation

The sample size was calculated using an Epitools calculator with a group sample ratio of 1, a 95% confidence level, a significance level of p at 0.05, and a desired power of 90%. A sample size of 10 in each group with a total sample size of 20 distributed equally in both groups.

Sample preparation

**Figure 1:**
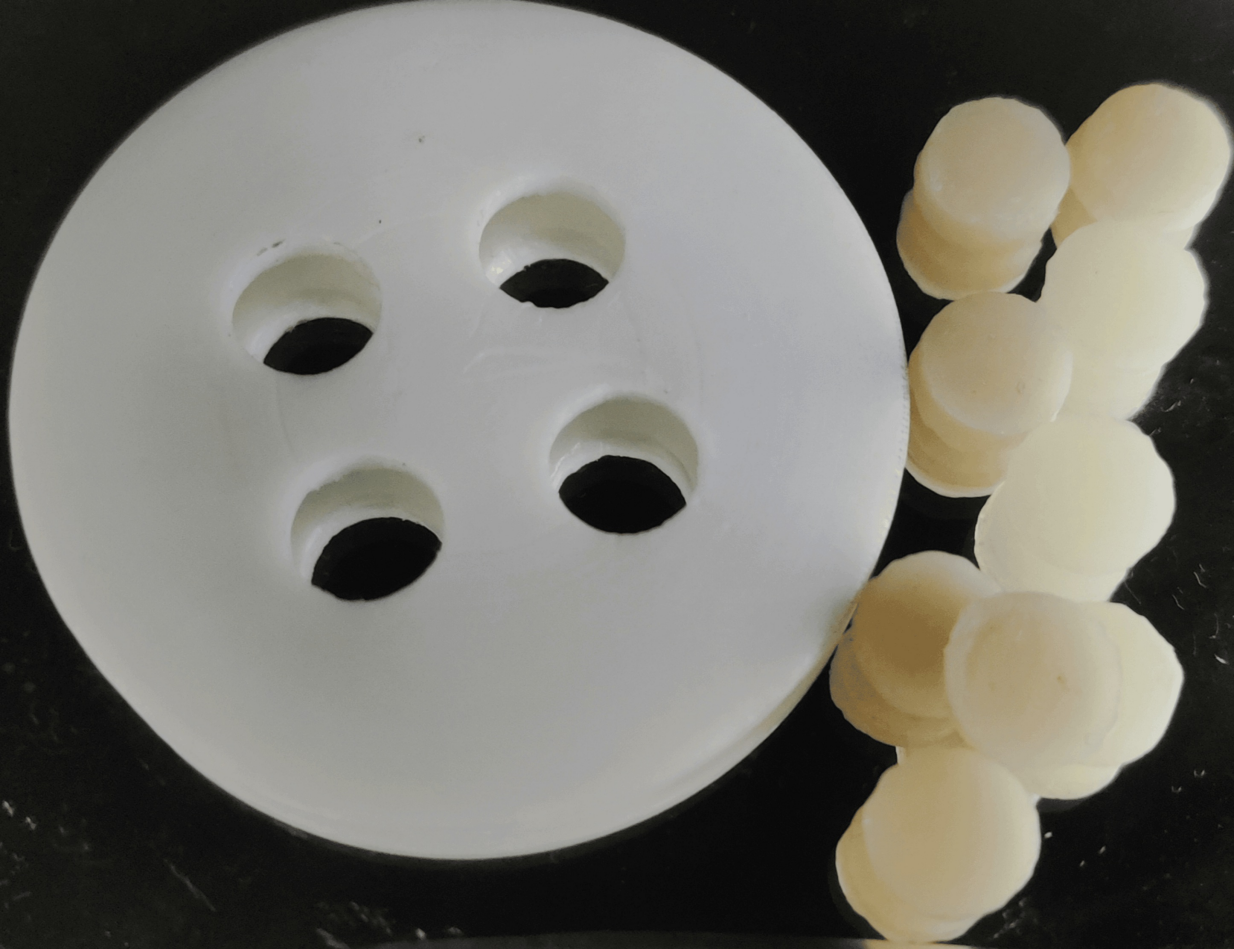
Teflon mold and composite resin samples

Ten single-shade composite resins and 10 multi-shade composite resins were inserted into a Teflon mold (Figure 1) and compressed using a transparent strip beneath a glass slide. The specimens underwent light irradiation by means of a light-emitting diode curing unit (Bluephase N MC, Ivoclar Vivadent, Liechtenstein), which was directed through the slide. The specimens' thickness was measured using a digital caliper (Mitutoyo, Japan) and found to be 5 mm.

Surface roughness assessment and toothbrush simulation

**Figure 2:**
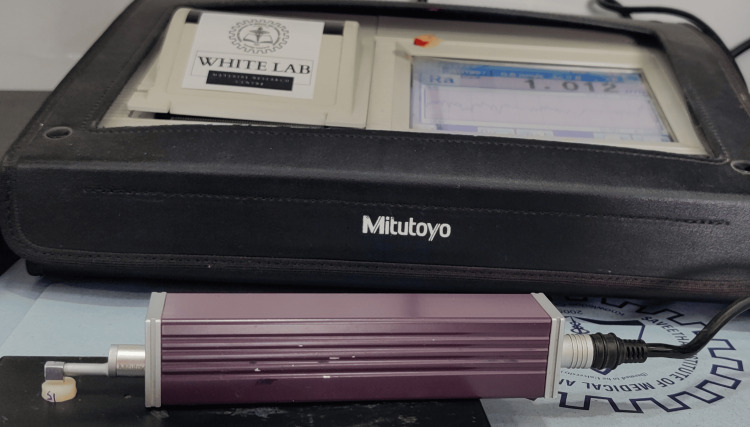
Evaluation of surface roughness using surface profilometer

**Figure 3:**
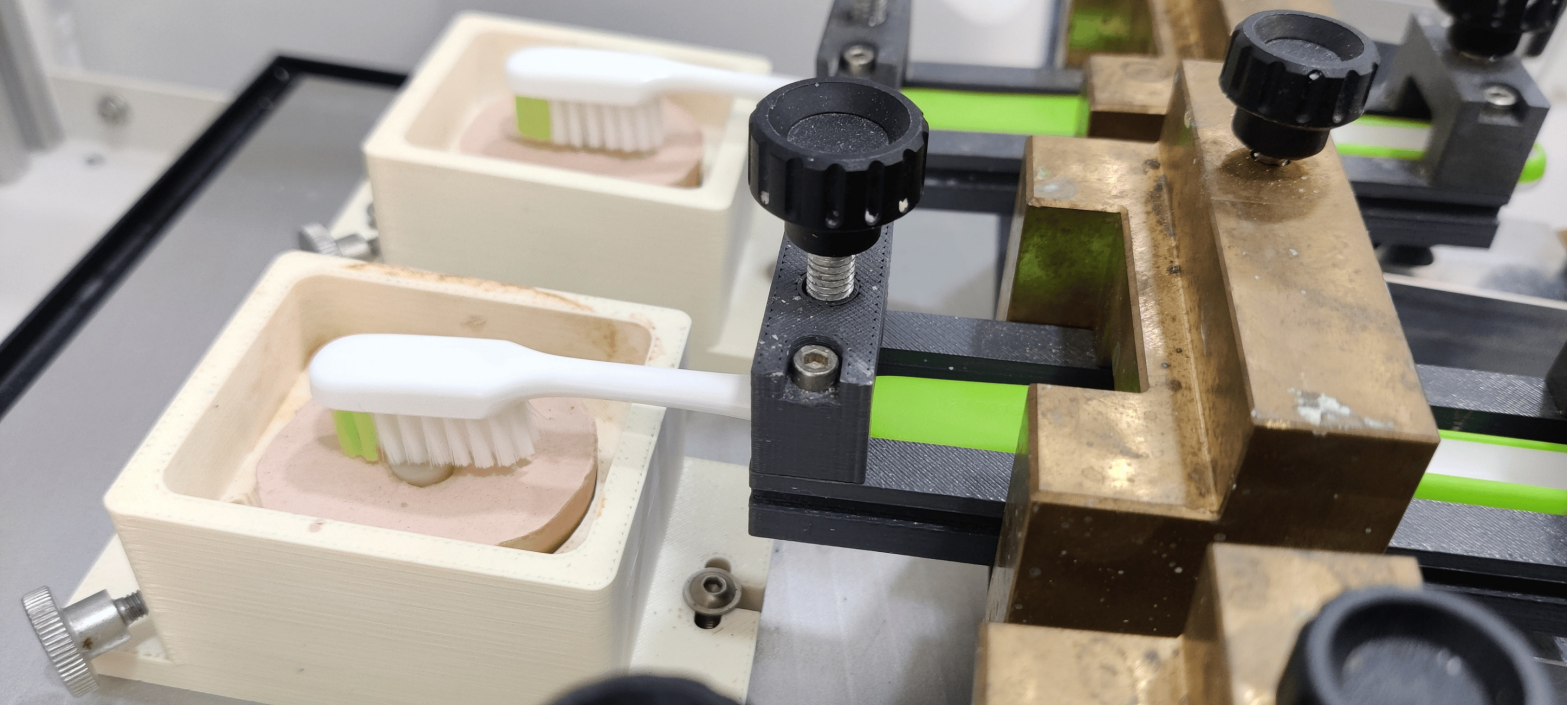
Toothbrush simulation

Pre-operative surface roughness values were evaluated using a surface profilometer (Mitutoyo, Japan) (Figure 2). Toothbrush abrasion was simulated in this study using a toothbrushing simulator (ZM3.8 SD Mechatronik, Germany) (Figure 3). A soft-bristled toothbrush (Sensodyne, USA) was fixed onto a holder and each sample was fixed on the sample holder. A low-abrasive toothpaste (Sensodyne, USA) was applied to these samples. All the resin molds were then subjected to a toothbrush simulator for 10,000 cycles, which will simulate one year of toothbrushing. The surface roughness of each of the samples after the toothbrush simulation was evaluated using a profilometer and recorded.

Color stability evaluation

**Figure 4:**
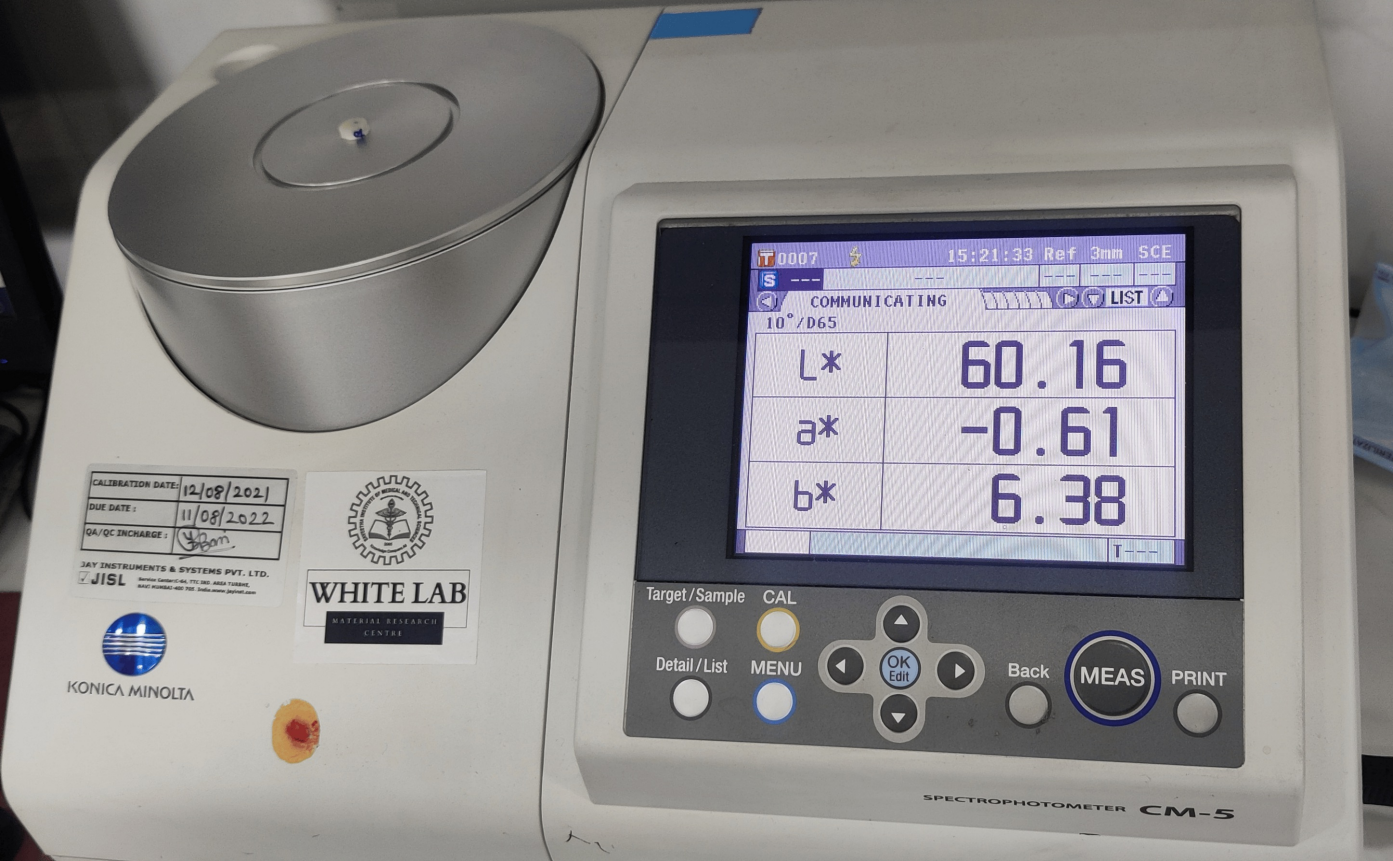
Evaluation of color using a spectrophotometer

**Figure 5:**
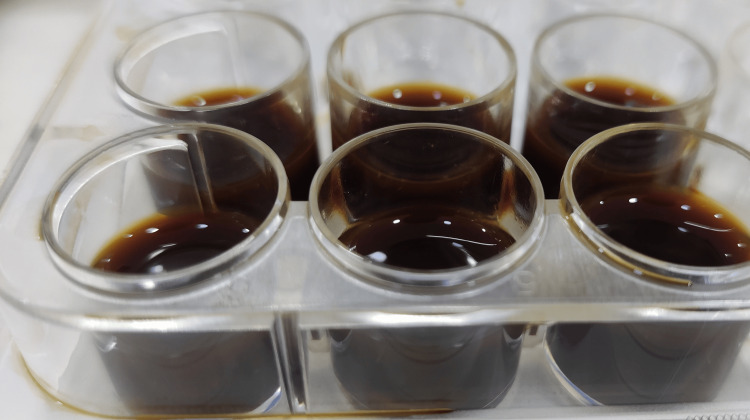
Immersed samples in beverages

All the resin molds were then evaluated for initial color (L*, a*, and b* values) using a spectrophotometer machine (Konica Minolta, Japan) (Figure 4). Five resin molds were then immersed in a beaker containing black coffee for seven days and five resin molds were immersed into a beaker containing aerated drink for seven days (Figure 5). Following this, the resin molds were re-evaluated for color stability using a spectrophotometer machine.

## Results

Statistical analysis

Data regarding surface roughness (µm) and color stability (ΔE) in multi-shade and single-shade composites was investigated for normality using the Shapiro-Wilk test and showed a normal distribution. Descriptive statistics were derived as mean and standard deviation. The surface roughness (µm) and color stability (ΔE) between multi-shade and single-shade composites were analyzed using an independent t-test. Pre- and post-surface roughness intervened using a toothbrush simulator in multi-shade and single-shade composites were analyzed using a paired t-test. Color stability (ΔE) of multi-shade and single-shade composites after seven days of immersion in coffee and Coca-Cola were analyzed using an independent t-test. The level of statistical significance was determined at p<0.05.

The intragroup comparison of surface roughness between multi-shade and single-shade composites pre-operatively and post-operatively shows that for both multi-shade and single-shade composites, there was an increase in surface roughness post-operatively and the values were statistically significant (Table 1).

**Table 1:** Intragroup comparison of surface roughness in multi-shade and single-shade composite ^*^Statistically significant (p<0.05).

Groups	Surface roughness (µm)	n	Mean+SD	Paired differences				
				Mean+SD	95% confidence interval of the difference		df	Paired t-test value (p-value)
					Lower	Upper		
Multi-shade composite	Pre-op	10	1.42+0.08	-1.00+0.44	-1.31	-0.68	9	-7.111 (0.000)*
	Post-op	10	2.42+0.50					
Single-shade composite	Pre-op	10	1.72+0.11	-1.23+0.20	-1.37	-1.08	9	-19.221 (0.000)*
	Post-op	10	2.95+0.23					

**Figure 6:**
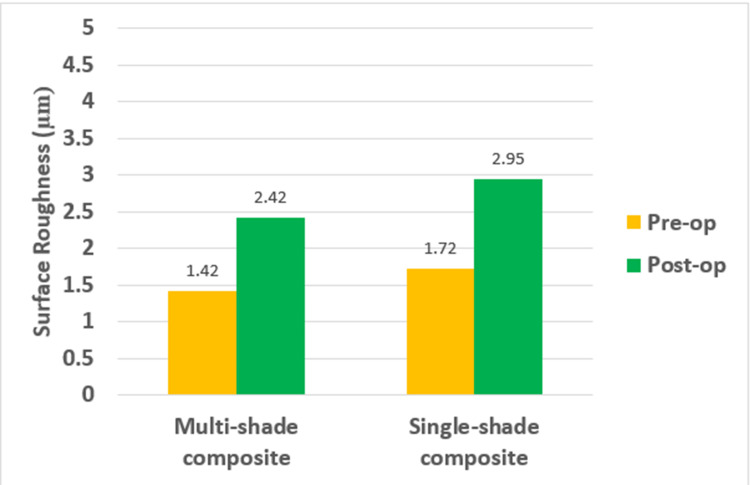
Surface roughness pre- and post-subjecting it to a toothbrush simulator in multi-shade and single-shade composites

The intergroup comparison of surface roughness between multi-shade and single-shade composites pre-operatively and post-operatively shows that the surface roughness values were highest for single-shade composites of both pre-operatively and post-operatively, with the values being statistically significant (Table 2, Figure 6).

**Table 2:** Intergroup comparison of surface roughness between multi-shade and single-shade composite ^*^Statistically significant (p<0.05).

Surface roughness (µm)	Groups	n	Mean+SD	Mean difference	95% confidence interval of the difference		df	Independent t-test value (p-value)
					Lower	Upper		
Pre-op	Multi-shade composite	10	1.42+0.08	-0.30	-0.39	-0.20	18	-6.407 (0.000)*
	Single-shade composite	10	1.72+0.11					
Post-op	Multi-shade composite	10	2.42+0.50	-0.53	-0.90	-0.16	18	-3.047 (0.007)*
	Single-shade composite	10	2.95+0.23					

The color stability of the multi-shade and single-shade composites after immersion in coffee and Coca-Cola for seven days shows that there is increased discoloration following immersion in coffee for both the composites and that the values were statistically significant (Table 3).

**Table 3:** Color stability in multi-shade and single-shade composite after immersion in coffee and Coco-Cola for seven days ^*^Statistically significant (p<0.05).

Groups	Immersion	n	Mean+SD	Mean difference	95% confidence interval of the difference		df	Independent t-test value (p-value)
					Lower	Upper		
Muti-shade composite	Coffee	5	5.38+0.74	4.87	4.10	5.64	8	14.642 (0.000)*
	Coco-Cola	5	0.50+0.05					
Single-shade composite	Coffee	5	7.14+1.34	6.36	4.97	7.75	8	10.571 (0.000)*
	Coco-Cola	5	0.77+0.06					

**Figure 7:**
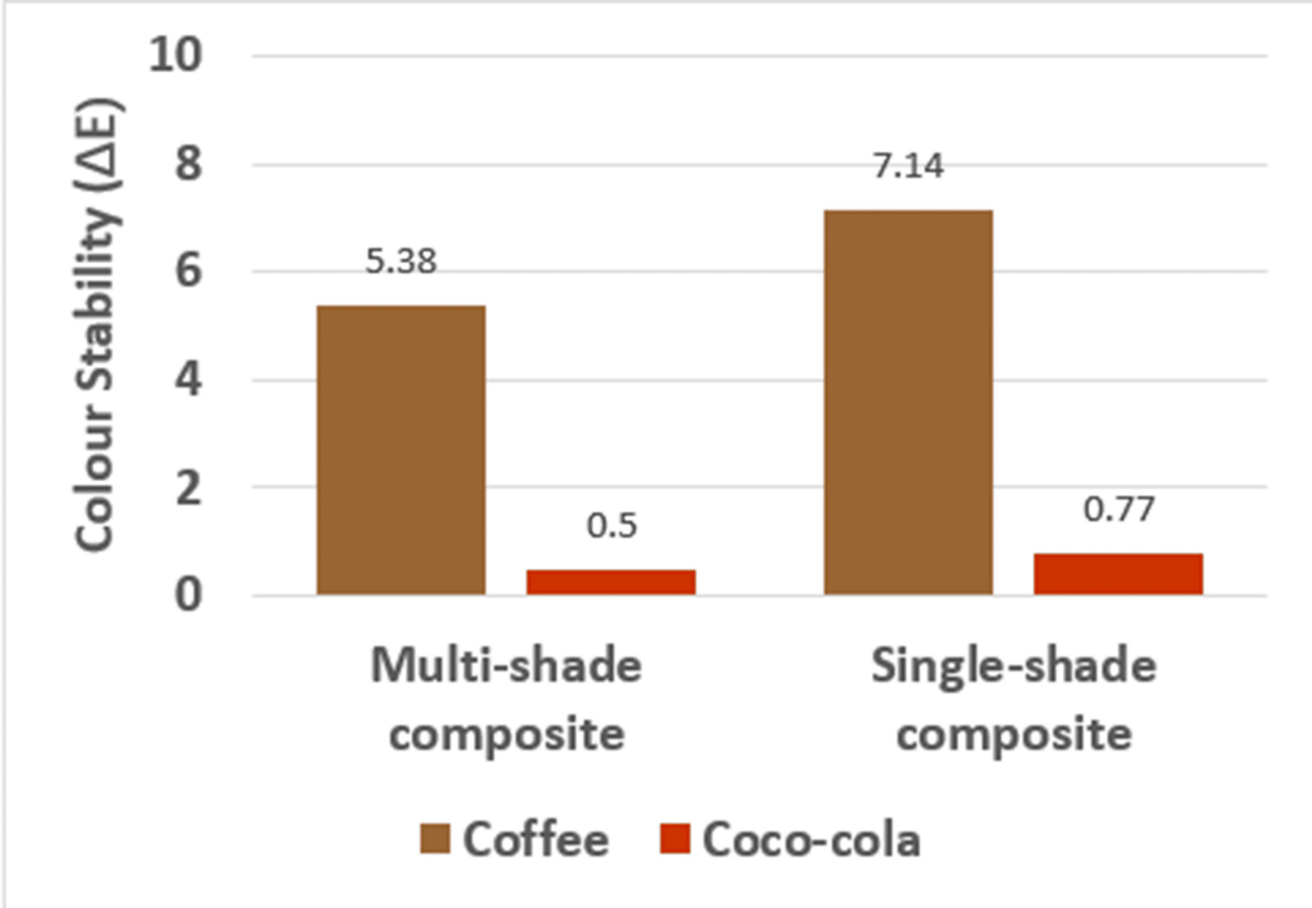
Color stability after immersion in coffee and Coco-Cola for seven days

The intergroup comparison for color stability between multi-shade and single-shade composite shows that there is increased discoloration for the single-shade composite with the values being statistically significant (Table 4 and Figure 7)

**Table 4:** Intergroup comparison of color stability between multi-shade and single-shade composite after immersion in coffee and Coco-Cola for seven days ^*^Statistically significant (p<0.05).

ΔE values	Groups	n	Mean+SD	Mean difference	95% confidence interval of the difference		df	Independent t-test value (p-value)
					Lower	Upper		
After immersion in coffee (7 days)	Multi-shade composite	5	5.38+0.74	-1.76	-3.34	-0.17	8	-2.561 (0.034)*
	Single-shade composite	5	7.14+1.34					
After immersion in Coco-Cola (7 days)	Multi-shade composite	5	0.50+0.05	-0.27	-0.35	-0.18	8	-6.999 (0.000)*
	Single-shade composite	5	0.77+0.06					

## Discussion

In dentistry, resin-based composite materials are frequently employed as direct tooth-colored restorative materials [9]. The challenge for clinicians is to replicate the color of natural dentition in response to the heightened aesthetic demands [10]. Most clinicians use multi-shade composite resins in their day-to-day practice. The multi-shade resin composites are labeled with letters to represent the hue and numbers to denote chroma and value [11]. However, the existence of different shades can make the process of matching shades more complex and result in higher costs and more time spent chairside [12].

A single-shade resin composite, also known as a universal shade, has been developed to supplant a variety of shades. This composite resin contains nanofillers (nanomers) and nanomer groups (nanoclusters), which are known to offer enhanced color harmony with tooth tissues. These composite resins offer shade matching for all tooth colors by utilizing a single shade that has a chameleon or blending effect, which allows the composite to match the color of its adjacent teeth [1]. Omnichroma is one such monochromatic resin composite. It uses supra nanofillers of 260 nm, and the phenomenon of structural coloration arises from the interplay between light and the microstructure of a substance [13]. It has established itself as one of the most effective monochromatic composites in clinical practice. Extensive research has been done on this material, and it is generally thought to be one of the best materials for effective color-matching [1].

Color is an important factor in the aesthetics of a composite restoration. The color stability of restoration is determined by the composition of the resin matrix, the size of filler particles, the extent of polymerization, and the presence of coloring additives [14,15]. Also, surface roughness is said to influence color, as resin composites with a rough surface exhibited a lower degree of chromaticity than those with a smooth surface [2]. After the placement of a composite restoration in the mouth, a series of intricate processes occur, ultimately resulting in the degradation of the restoration. Toothbrush abrasion is said to be the most common cause of wear. Toothbrush abrasion refers to the wearing away of tooth enamel and gum tissue caused by excessive or aggressive brushing with a toothbrush [16].

In this study, both the single-shade composite resin Omnichroma and multi-shade composite resins were tested first for their surface roughness. The surface roughness (Ra) is defined as the average deviation of the profile from the center line of the surface [17]. The measurement of roughness can be conducted using various methods; however, the most frequently employed in dentistry is the surface roughness (Ra) value. In this study, surface roughness was measured using a surface profilometer, which used a diamond stylus that is vertically displaced to make contact with a sample and is laterally displaced across the sample for a set distance and with a specified contact force. It was noted that, after toothbrush abrasion, all the evaluated materials in this study showed a significant increase in surface roughness [2].

Spectrophotometers have been increasingly used to evaluate tooth color in recent years. The clinical spectrophotometer device provides more objective measurements [18]. Commission Internationale de l´Eclairage (CIELAB) is calculated using the formula ΔEab using the color change values L*, a*, and b* in the materials [19]. Therefore in this study, Konica Minolta CM5 spectrometer was utilized in the basic shade mode to accurately assess the color of the samples.

The results of this study showed that there is an increase in surface roughness and color changes for the single-shade composite when compared with the multi-shade composite. However, previous research has shown that composites with nanofillers show lesser surface roughness when compared with micro-hybrid composites [3]. The difference in this study could have been due to the roughness caused during the packing of the material in the resin mold.

Research indicates that composites with a primary monomer composition of bisphenol A-glycidyl methacrylate (Bis-GMA) exhibit lower water absorption compared to composites containing triethylene glycol dimethacrylate (TEGDMA). The multi-shade composite (spectrum), which was used in this study, has Bis-GMA as its primary monomer compared to the single-shade composite (Omnichroma), which had TEGDMA as the primary monomer [20]. The variation in color was directly associated with the absorption of water, which in turn was associated with the specific composition of the base monomer. Increased resin content in dental materials results in higher water absorption, which in turn leads to hydrolytic degradation. This degradation can lead to changes in the material's physical and optical characteristics. The polymer matrix's ability to absorb water has been discovered to change colors by either destroying the connection between the matrix and filler or causing the filler to undergo hydrolytic breakdown [19]. This could have been the reason for increased color changes in single-shade composite compared with the multi-shade composite resin.

Furthermore, the translucency of the material increases as a result of the disparity in the refractive index between the composite resins prior to and following polymerization. Single-shade resin composites are reported to possess enhanced translucency, allowing them to effectively match a diverse array of hues [20]. The drop in the translucency is believed to result in increased color changes in single-shade composites, which may also have been the reason for discoloration [20].

The aerated drink’s low pH might damage the surface integrity of resin composite materials [21]. However, this study did not produce as much discoloration as the samples immersed in black coffee possibly due to the colorants that are present in coffee. During the process of coffee roasting, there is the formation of brown-colored nitrogenous compounds with high molecular weight, known as melanoidins [22]. These compounds are principally responsible for the coloring observed in coffee. Additional pigments like tannin or caffeine add to discoloration by thoroughly infiltrating the composite matrix [23,24].

This study was done in vitro, wherein stains were formed on both surfaces of the restorative material. Nevertheless, in practical application, only the external layer of the resin composite is subjected to solutions, and the beverages consumed can be diluted by saliva [20]. Another limitation of this study was that the chameleon effect of the single-shade composite can not be achieved in an in-vitro condition. Additionally, the disparity in surface roughness between the composites in this investigation and the prior ones may have posed a restriction.

## Conclusions

Based on this investigation, it has been suggested that conducting clinical trials on the color alteration of single-shade resin composites would be advantageous. The results of this study show that there is a statistical difference between the single-shade composite and the multi-shade composite when it comes to surface roughness and discoloration. Thus, it can be concluded that even though single-shade composites are said to offer ease of shade selection, their longevity in the oral cavity is lesser compared to multi-shade composites as they have a greater tendency for discoloration in beverages compared to multi-shade composite resins.
